# *In Vitro* Fermentation of NUTRIOSE^®^ FB06, a Wheat Dextrin Soluble Fibre, in a Continuous Culture Human Colonic Model System

**DOI:** 10.1371/journal.pone.0077128

**Published:** 2013-10-24

**Authors:** Mark R. Hobden, Agustin Martin-Morales, Laetitia Guérin-Deremaux, Daniel Wils, Adele Costabile, Gemma E. Walton, Ian Rowland, Orla B. Kennedy, Glenn R. Gibson

**Affiliations:** 1 Department of Food and Nutritional Sciences, The University of Reading, Reading, United Kingdom; 2 Roquette, Lestrem, France; Charité-University Medicine Berlin, Germany

## Abstract

Wheat dextrin soluble fibre may have metabolic and health benefits, potentially acting via mechanisms governed by the selective modulation of the human gut microbiota. Our aim was to examine the impact of wheat dextrin on the composition and metabolic activity of the gut microbiota. We used a validated *in vitro* three-stage continuous culture human colonic model (gut model) system comprised of vessels simulating anatomical regions of the human colon. To mimic human ingestion, 7 g of wheat dextrin (NUTRIOSE^®^ FB06) was administered to three gut models, twice daily at 10.00 and 15.00, for a total of 18 days. Samples were collected and analysed for microbial composition and organic acid concentrations by 16S rRNA-based fluorescence *in situ* hybridisation and gas chromatography approaches, respectively. Wheat dextrin mediated a significant increase in total bacteria in vessels simulating the transverse and distal colon, and a significant increase in key butyrate-producing bacteria *Clostridium* cluster XIVa and *Roseburia* genus in all vessels of the gut model. The production of principal short-chain fatty acids, acetate, propionate and butyrate, which have been purported to have protective, trophic and metabolic host benefits, were increased. Specifically, wheat dextrin fermentation had a significant butyrogenic effect in all vessels of the gut model and significantly increased production of acetate (vessels 2 and 3) and propionate (vessel 3), simulating the transverse and distal regions of the human colon, respectively. In conclusion, wheat dextrin NUTRIOSE^®^ FB06 is selectively fermented *in vitro* by *Clostridium* cluster XIVa and *Roseburia* genus and beneficially alters the metabolic profile of the human gut microbiota.

## Introduction

The increasing worldwide prevalence of obese and overweight individuals is a major public health concern [1]. Obesity, and more specifically the accretion of excess adipose tissue, is associated with elevated chronic systemic low-grade inflammation and increased risk of metabolic diseases, such as type 2 diabetes and cardiovascular disease (CVD) [2]. The pathogenesis of these conditions is attributable to a complex interaction between genetic, metabolic, environmental and behavioural factors, however the specific contribution of each of these determinants is not fully understood [3]. The composition and metabolic activity of microbial inhabitants of the human gut has recently been acknowledged as an environmental factor that may influence the development of obesity and associated metabolic diseases [4,5]. 

The human gut microbiota is a diverse ecosystem comprising of up to 100 trillion archaeal and bacterial cells. Any of between 1,000 - 1,150 bacterial species may reside in the gut, with most individuals harbouring at least 160 different species. Although over 90% of gut bacteria belong to Bacteroidetes and Firmicutes, the specific composition of the gut microbiota, at the phylum, genus and species level, is highly individual and affected by various factors, including adiposity [6]. Obesity and diet-induced weight gain have been associated with a modified microbial composition, with some studies [7-10], but not all [11-13], providing evidence for an altered Firmicutes-Bacteroidetes ratio. Reduced numbers of *Eubacterium rectale-Clostridium coccoides* cluster*, Bifidobacterium* spp.*, Lactobacillus* spp. and *Roseburia* spp., have been observed in mice subjected to an obesogenic diet [14-17]. Furthermore, a recent rodent model study by Liou et al., has provided the first empirical evidence that changes in the gut microbiota may contribute towards reduced host weight and adiposity [18].

The gut microbiota has a major influence on host metabolism, with microbe-host interactions connecting with organs, including the gut, liver, adipose tissue, muscle and brain. Accordingly, dietary modulation of the composition and activity of the gut microbiota, with food ingredients such as prebiotics, has been highlighted as a potential target for obesity and metabolic diseases [5]. Prebiotics are defined as ‘selectively fermented dietary ingredients that result in specific changes in the composition and/or activity of the gastrointestinal microbiota, thus conferring benefit(s) upon host health’ [19]. Woods and Gorbach included in this definition an increase in beneficial bacteria and/or a decrease in harmful types, a reduction in intestinal pH, production of SCFA and changes in bacterial enzyme concentrations [20]. The fermentation of a food ingredient by the gut microbiota is dependent on its physicochemical structure [4]. Thus far, most attention has focused on the prebiotic potential of soluble fibres, in particular non-digestible oligosaccharides such as inulin-type fructooligosaccharides (FOS) and trans-galactooligosaccharides (TOS), with the daily intake of 2.75 - 20 g shown to positively alter gut microbial composition after a short feeding period [21]. Other soluble fibres, including resistant dextrins (wheat or starch), glucans, gums and pectins are also increasingly recognised as having prebiotic potential. The intake of wheat dextrin (WD) and FOS has been shown to have satiogenic and weight management benefits, possibly attributable to elevated synthesis of anorexigenic gut hormones (peptide YY (PYY) and glucagon-like peptide 1 (GLP-1)) and decreased synthesis of the orexigenic gut hormone, ghrelin [22-24]. Furthermore, TOS has recently been found to beneficially impact on metabolic markers of immune function, systemic inflammation and blood glucose regulation [25]. The mechanisms responsible for these effects remain to be elucidated, however up regulation of short-chain fatty acids (SCFA), acetate, propionate and butyrate, which are key metabolic end-products of bacterial fermentation, may play pivotal roles [26].

NUTRIOSE^®^ (NUTRIOSE^®^ FB06, Roquette, France) is a non-viscous WD with a total fibre content of ~ 85% and a mono- and disaccharide content of ≤ 0.5% [27]. NUTRIOSE^®^ has a structure of linear and branched glucosidic linkages that make it resistant to hydrolysis in the small intestine and consequently available for bacterial fermentation in the human large gut [28]. NUTRIOSE^®^ induces a low glycaemic response and is well tolerated by the human digestive system, even at high doses [29,30]. Emerging evidence indicates that NUTRIOSE^®^ has prebiotic potential, however most of the studies have been done in animal models, which have a different microbiota from that of humans, or have investigated a limited number of bacterial groups in humans. Nevertheless these studies have demonstrated that the intake of NUTRIOSE^®^ may modulate gut microbial ecology, with evidence for increased faecal counts of *Bacteroides* and *Lactobacillus* spp., reduced *Clostridium perfingens*, increased total SCFA production, elevated α- and β- glucosidase activity and decreased faecal pH [29-32]. Furthermore, NUTRIOSE^®^ has been shown to have promising effects on energy metabolism, appetite regulation and weight management [33-36]. In a recent human intervention study by Guerin-Deremaux et al., the daily intake of either 14 g, 18 g, or 24 g of NUTRIOSE^®^, over a period of nine weeks, was found to increase perceived feelings of satiety and subsequently lead to a reduction in energy intake, bodyweight and percentage body fat in a group of overweight males [34]. Preliminary evidence also suggests that NUTRIOSE^®^ may improve indices of lipid and glucose homeostasis, however additional work is warranted to verify these findings [37]. 

The aim of the present study was to examine more fully the impact of WD fermentation on gut microbial ecology and metabolic end products of microbial fermentation in distinct anatomical regions of the human colon using an *in vitro* three-stage continuous culture human colonic model (gut model) system. This system has been used extensively at our institution and provides a controlled and steady-state environment in which to study the composition and metabolic activities of the gut microbiota in relation to external perturbations, such as with the administration of food ingredients [38]. The intake of 14 g/day NUTRIOSE^®^ was the lowest dose to significantly increase satiety, reduce energy intake and improve body composition in previous investigations by Guerin-Deremaux and colleagues [34,36]. Accordingly, this dosage was chosen for the present study and was administered in two equal doses each day to mimic human ingestion. The impact of WD fermentation on the proliferation of a selection of the main bacterial groups of the gut microbiota, including groups with purported benefits or detriments to health, was assessed using 16S rRNA-based fluorescence in situ hybridisation (FISH) and organic acid production analysed by gas chromatography (GC). 

## Materials and Methods

### Three-stage continuous culture colonic model (gut model) system

Three gut models, each comprising of a cascade of three glass fermenters connected in series that simulate the different physical and nutritional characteristics of the proximal (V1), transverse (V2) and distal colon (V3), were implemented under conditions previously detailed by Macfarlane et al. [38]. Gut models were inoculated with faecal samples from healthy human donors, all of whom had no previous history of gastrointestinal disorders and had not received antibiotics or pre/probiotics for at least three months prior to the study. All faecal donors had the experimental procedure explained to them and were given the opportunity to ask questions. All donors then provided verbal informed consent for the use of their faeces in the study. The University of Reading research ethics committee exempted this study from review because no donors were involved in any intervention and waived the need for written consent due to the fact the samples received were not collected by means of intervention. Equilibrium of the system, steady state 1 (SS1), was reached after eight full turnovers at 15, 16 and 17 days. Thereafter, the test product, NUTRIOSE^®^, was administered into V1 in 7 g doses at 10.00 and 15.00 each day until the second steady state (SS2) had been reached at 33, 34, and 35 days. Samples (1 mL) were collected from all vessels of the colonic system and centrifuged at 13,000 x g for 10 min to remove all particulate matter. A shortened GC method was used to determine the stabilisation of SCFA concentrations over three consecutive days and confirm steady state (SS1 and SS2) [39]. Further samples were stored at -18 °C for future analysis.

### *In vitro* enumeration of bacterial populations by FISH

Enumeration of bacterial populations was performed by FISH using synthetic oligonucleotide probes designed to target specific diagnostic regions of 16S rRNA and labelled with the fluorescent Cy3 dye (Sigma Aldrich Ltd., Poole, Dorset, UK), as previously described [40]. As detailed in Table 1, probes were used for the determination of total bacteria, bifidobacteria, lactobacilli/enterococci, *Bacteroides*, *Clostridium perfringens/histolyticum* subgroup, *Clostridium* cluster XIVa, *Clostridium* cluster I and II, *Roseburia* genus and *Atopobium - Coriobacterium*.

**Table 1:** Probes used for bacterial enumeration by FISH.

**Probe name**	**Sequence (5’ to 3’)**	**Target species**	**Hybridization- Washing Temperature (°C)**	**Reference**
**Ato291**	GGTCGGTCTCTCAACCC	*Atopobium*, *Colinsella*, *Olsenella* and *Eggerthella* spp.; *Cryptobacterium curtum*; *Mycoplasma equigenitalium* and *Mycoplasma elephantis*	50 - 50	[54]
**Bac303**	CCAATGTGGGGGACCTT	Most *Bacteroides sensu stricto* and *Prevotella* spp.; *all Parabacteroides*; *Barnesiella viscericola and Odoribacter splanchnicus*	46 - 48	[55]
**Bif164**	CATCCGGCATTACCACCC	Most *Bifidobacterium* spp. and *Parascardovia denticolens*	50 - 50	[56]
**Chis150**	TTATGCGGTATTAATCTYCCTTT	Most members of *Clostridium cluster I*; all members of *Clostridium cluster II; Clostridium tyrobutyricum; Adhaeribacter aquaticus* and *Flexibacter canadensis* (family *Flexibacteriaceae*)*;* [Eubacterium] *combesii* (family *Propionibacteriaceae*)	50 - 50	[57]
**Erec482**	GCTTCTTAGTCARGTACCG	Most members of *Clostridium cluster XIVa; Syntrophococcus sucromutans*, [Bacteroides] *galacturonicus* and [Bacteroides] *xylanolyticus, Lachnospira pectinschiza* and *Clostridium saccharolyticum*	50 - 50	[57]
**Lab158**	GGTATTAGCAYCTGTTTCCA	Most *Lactobacillus, Leuconostoc* and *Weissella* spp.; *Lactococcus lactis*; all *Vagococcus, Enterococcus, Melisococcus, Tetragenococcus, Catellicoccus, Pediococcus* and *Paralactobacillus* spp*.*	50 - 50	[58]
**Rrec584**	TCAGACTTGCCGYACCGC	*Roseburia - Eubacterium rectale* (a component of cluster XIVa)	50 - 50	[59]
**Eco1531**	CACCGTAGTGCCTCGTCATC	*Escherichia coli*	37 - 37	[59]
**EUB338**	GCTGCCTCCCGTAGGAGT	Total bacteria	46 - 48	[60]
**EUB338II**	GCAGCCACCCGTAGGTGT	Total bacteria	46 - 48	[60]
**EUB338II**	GCTGCCACCCGTAGGTGT	Total bacteria	46 - 48	[60]

### Organic acid determination by GC

Samples were extracted and derivatised as previously described by Richardson et al. [41]. Briefly, aliquots of 1 mL of supernatant were transferred into glass tubes, followed by the addition of 50 µL of internal standard (100 mM; 2-ethylbutyric acid), 500 µL of concentrated hydrochloric acid (HCl) and 2 mL of diethyl ether (Sigma Aldrich Ltd., Poole, Dorset, UK). Samples were then vortexed for 1 min before centrifugation at 3,000 x g for 10 min. The top ether layer was transferred from each tube into clean glass tubes. A second extraction step was then completed using a further 1mL of diethyl ether. The diethyl layer was again collected and pooled with the layer from the first extraction. Aliquots of 400μL of this pooled extract were transferred into glass vials, alongside 50 μL of *N*-methyl-*N*-*t*-butyldimethylsilyltrifluoroacetamide (Cheshire Sciences, Chester, UK). The samples were then incubated at 80°C in a water bath for 20min and left at room temperature for 48 h to allow for the complete derivatisation of lactic acid. 

The derivatized samples were run on a 5890 SERIES II Gas Chromatograph (Hewlett Packard, UK) with flame ionization detector, using an Rtx-1 10m×0.18mm column coated with a 0.20μm coating (Crossbond 100% dimethyl polysiloxane; Restek, Buckinghamshire, UK). Injector and detector temperature were set at 275°C and the column temperature was programmed from 63°C for 3 min to 190°C at 10°C/min^-1^ and held at 190 °C for 3 min. Helium was used as the carrier gas (flow rate 1.2 mL/min; head pressure 90 MPa). External standards contained (mM): sodium formate, 10; acetic acid, 30; propionic acid, 20; iso-butyric acid, 5; n-butyric acid, 20; iso-valeric acid, 5; n-valeric acid, 5; sodium lactate, 10; sodium succinate, 20. Chemstation B.03.01 (Agilent Technologies, Cheshire, UK) was used for calibration and calculation of the internal response factor for quantification of peak areas within samples.

### Statistical analysis

All statistical tests were performed on GraphPad Prism 6.0 (GraphPad Software, La Jolla, CA, USA). One-way AVOVA tests were used to compare SS1 and SS2 data for bacterial counts and organic acid concentrations. Where significant differences were identified, *post hoc* analysis was performed using Tukey multiple comparison tests. Statistical significance was accepted at *P* < 0.05 for all analyses.

## Results

### The impact of WD on the human gut microbiota

**Figure 1:**
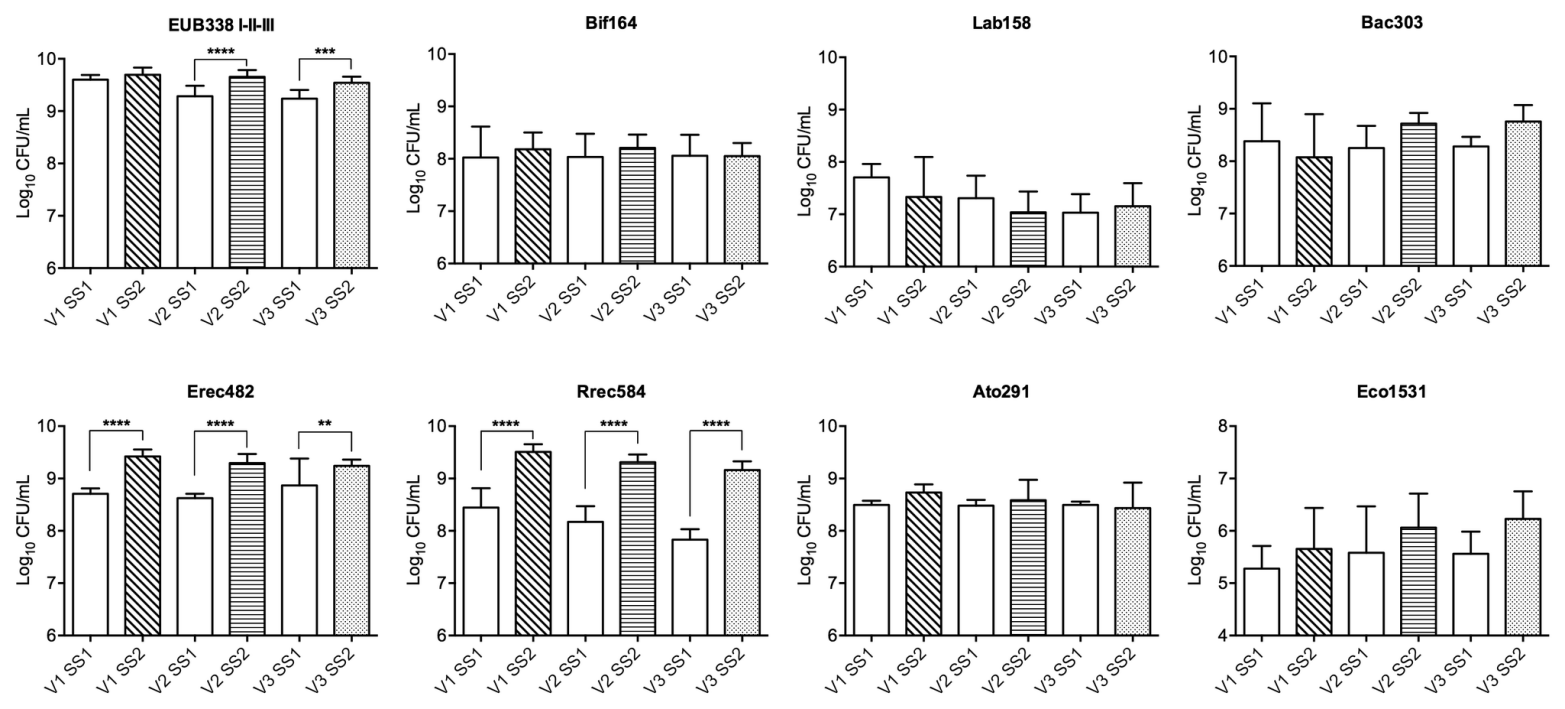
Bacterial populations. Bacterial populations in the three different vessels (V1, V2 and V3) of the gut models before (SS1) and after (SS2) WD treatment. Data presented as means of the three gut models ± standard deviations. SS1 and SS2 are calculated as mean values over three consecutive days. ** *P* < 0.01, *** *P* < 0.001, **** *P* < 0.0001, significantly different from SS1.

Average bacterial counts, as enumerated by FISH, are displayed in Figure 1 and expressed as log CFU/ml ± standard deviations. Following administration of WD, total bacterial populations significantly increased by 0.37 log_10_ in V2 simulating the transverse colon (*P* < 0.0001) and 0.30 log_10_ in V3 simulating the distal colon (*P* < 0.001). No significant differences for total bacteria were found in V1, simulating the proximal colon. Concentrations of *Clostridium coccoides* – *Eubacterium rectale* significantly increased by 0.71 log_10_ in V1 (*P* < 0.0001), 0.67 log_10_ in V2 (*P* < 0.0001) and 0.37 log_10_ in V3 (*P* < 0.01). Furthermore, concentrations of *Roseburia - E. rectale* significantly increased by 1.07 log_10_ in V1 (*P* < 0.0001), 1.14 log_10_ in V2 (*P* < 0.0001) and 1.33 log_10_ in V3 (*P* < 0.0001). No significant differences were found for the other bacterial groups analysed, including *Bifidobacterium* spp.*, Lactobacillus* - *Enterococcus*., *Bacteroides - Prevotella, Atopobium - Coriobacterium and Escherichia coli*. There were trends for increases in *Bifidobacterium* spp. and *Escherichia coli* in all vessels, however these did not reach significance. Counts for the *Clostridium histolyticum* group were below the detection level (data not shown).

### Organic acid production

Relative concentrations of organic acids, as determined by GC, are reported in Table 2 and expressed as mM ± standard deviations. Fermentation of WD mediated a significant 19.45 mM increase in butyrate in V1 (*P* < 0.0001), 30.38 mM increase in V2 (*P* < 0.001) and 27.01 mM increase in V3 (*P* < 0.01) of the gut models. In V1, simulating the proximal colon, WD administration resulted in a significant 9.82 mM reduction in acetate (*P* < 0.01) and 14.71 mM reduction in propionate (*P* < 0.05) concentrations. In V2, simulating the transverse colon, acetate concentrations significantly increased by 16.11 mM (*P* < 0.01), whereas propionate concentrations did not change. In V3, simulating the distal colon, acetate concentrations significantly increased by 29.16 mM (*P* < 0.001) and propionate concentrations significantly increased by 14.64 mM (*P* < 0.01). Concentrations of formate, iso-valeric, valeric and lactate were below the detection limit.

**Table 2:** SCFA concentrations.

	Acetate			Propionate			Butyrate	
	SS1	SS2		SS1	SS2		SS1	SS2
Vessel 1	31.92 ± 6.12	22.10 ± 4.28 **		33.82 ± 7.65	19.12 ± 8.01 *		30.75 ± 3.10	50.20 ± 2.47 ****
Vessel 2	45.77 ± 3.56	61.88 ± 7.65 **		41.02 ± 5.53	40.87 ± 6.70		34.78 ± 6.11	65.16 ± 8.72 ***
Vessel 3	50.82 ± 8.37	79.97 ± 10.42 ***		43.15 ± 6.81	57.80 ± 8.24 **		36.98 ± 6.01	63.99 ± 9.43 **

Mean SCFA concentrations reported as mM ± standard deviations for the three vessels of the gut models before (SS1) and after (SS2) WD treatment. SS1 and SS2 are calculated as mean values over three consecutive days. * *P* < 0.05, ** *P* < 0.01, *** *P* < 0.001, **** *P* < 0.0001, significantly different from SS1.

## Discussion

The aim of the present study was to extend knowledge of the impact of WD on the microbial ecology of the human gut. A validated gut model system was used to establish the effects of *in vitro* fermentation of WD on the proliferation of a range of known bacterial groups. These included the purported health-promoting *Bifidobacterium* and *Lactobacillus* genera, but also other bacteria increasingly recognised for having important roles in the human colon, including *Bacteroides* and *Roseburia* genus. In addition, metabolic end products of microbial fermentation, including SCFAs, acetate, propionate and butyrate, were analysed to elucidate potential mediators by which this novel food ingredient may exert metabolic and health benefits to the human host through modulation of the gut microbiota.

The provision of WD, at a dose of 14 g/day, resulted in marked increases in total bacterial populations in vessels simulating the transverse and distal colon. This increased proliferation of total bacteria is concurrent with findings from previous studies in rodents and humans [29-32]. To our knowledge, we have demonstrated for the first time that WD significantly increases the proliferation of Gram-positive *Clostridium* Cluster XIVa bacteria and *Roseburia* genus, as categorised by increased counts for *Clostridium coccoides* – *Eubacterium rectale* and *Roseburia - E. rectale* groups, respectively. These bacterial groups are major components of the human gut microbiota and have important roles in the fermentation and putrefaction of food-derived substances, resulting in the production of SCFAs and intestinal gases [42]. Furthermore, *Clostridium* Cluster XIVa and *Roseburia* genus are key butyrate producers, with ~ 80% of the butyrate-producing isolates originating in human faeces belonging to these groups [43]. Experimental work in rodents suggests that obesity is inversely correlated to numbers of *Clostridium* Cluster XIVa and *Roseburia* genus. Interestingly, these studies found that the provision of dietary fibres, chitin–glucan and wheat arabinoxylan, to mice subjected to an obesogenic diet ameliorated the reduction in these two bacterial groups [14,17]. Owing to the findings of the present study, it is feasible that the intake of WD may also have restorative effects on gut microbial populations of *Clostridium* Cluster XIVa and *Roseburia* genus in obese and overweight individuals. 

Whilst there was a trend for an increase in *Bifidobacterium* spp. across the gut model, neither populations of this genus nor *Lactobacillus* - *Enterococcus*., significantly increased in response to the provision of WD. This, in combination with previous findings, suggests that the physicochemical structure of WD may not confer selectivity to bifidobacteria, which has been shown to prefer oligosaccharide structures, including TOS and various forms of galacto-oligosaccharides [44]. Pasman et al. observed increases in lactobacilli in response to WD, however this was following the intake of a higher dose of 45 g/day [31], with a recent study by Lefranc-Millot et al. [30] failing to observe such an effect with lower dosages. Lefranc-Millot et al. did however find that the intake of 10 g/day WD increased numbers of *Bacteroides*, a genus of bacteria that may benefit the human host by preventing potential pathogens from colonising the gut. Furthermore, the administration of 8 g/day and 15 g/day reduced numbers of *Clostridium perfringens*, an opportunistic human pathogen [30]. In the current study *Clostridium perfringens* levels were below the detection threshold in all three human donors and subsequently we were unable to determine the impact of WD on this bacterial group. Whilst there was a trend for an increase in *Bacteroides* in the transverse and distal regions of the gut model, this did not reach significance. 

The observed increases in butyrate production across the three vessels of the gut model, together with increases in *Clostridium* cluster XIVa and *Roseburia* genus provide evidence for a butyrogenic effect of WD on gut microbial ecology. Moreover, the increasing magnitude of butyrate production through the gut model from V1-V3 strengthens support for a butyrogenic effect of WD in all regions of the human colon and eliminates the possibility that increased concentrations in the latter regions of the gut model were solely due to butyrate transiting through from a previous vessel in the system. Our findings support previous evidence from a rodent model that found that 14 days of WD supplementation increased faecal concentrations of butyrate, acetate and propionate [32]. Increased production of SCFA butyrate in the human large gut may have numerous health benefits. Firstly, butyrate is the preferred energy-providing substrate for colonocytes [45]. Secondly, butyrate has been shown to have a beneficial trophic effect on the gut epithelium [46]. Lastly, butyrate has been demonstrated to modulate colonic cell proliferation / apoptosis and may have protective properties against colon cancer and ulcerative colitis [47]. As the incidence of colon cancer and other colonic conditions, such as ulcerative colitis, is highest in the distal regions of the colon [48], the elevated production of butyrate and SCFAs, propionate and acetate, in V3 of the gut model is of considerable interest as regional differences in SCFA concentrations has implications for disease risk [49]. Furthermore, increased production of SCFAs indicates a preferential increase in carbohydrate fermentation rather than protein fermentation, which mainly occurs in the distal regions of the human colon due to reduced nutrient availability [50]. Rather than producing beneficial SCFAs and other organic acids, the breakdown of protein by proteolytic bacteria results in the formation of ammonia, phenols, indoles, thiols, amines and sulphides, which are potentially detrimental to health [51]. 

There is mounting evidence that prebiotics impact on appetite regulation and gut hormone release however the underlying mechanisms are not yet clear. Evidence from animal studies has recently highlighted the potential mechanistic importance of SCFA production by the gut microbiota. Findings from rodent models have demonstrated that butyrate and propionate, but not acetate, stimulate the secretion of anorexigenic hormones, GLP-1, PYY and gastric inhibitory polypeptide (GIP). These effects may be expressed through the activation of G-protein coupled receptors free-fatty acid receptor 2 and free-fatty acid receptor 3 by butyrate and propionate, however mechanisms independent of these receptors may also be involved [52]. Gut hormones are fundamental in the regulation of appetite but also in the control of glucose metabolism, with GIP involved in insulin dependent glucose uptake into muscle tissue and GLP-1 involved in insulin secretion from the pancreas [53]. In the present study, the WD mediated increases in butyrate and propionate production has identified possible metabolic mediators for any effects of WD fermentation on appetite regulation and energy metabolism. 

In conclusion, WD had a beneficial impact on gut microbial ecology, not only in V1 where administered, but persisting through all vessels of gut model system. Whilst not exerting a classical prebiotic effect, such as increased *Bifidobacterium* spp. or *Lactobacillus* spp., WD was selectively fermented by *Clostridium* Cluster XIVa and *Roseburia* genus, which are increasingly recognised as important health promoting components of the gut microbiota. Furthermore, observed increases in SCFA production, in particular butyrate and propionate, provide possible metabolic mediators for any effects of WD on host appetite regulation and energy metabolism. Future human intervention studies are required to establish the concomitant effects of WD on gut microbial ecology, appetite regulation and systemic markers of metabolism in order to elucidate underlying mechanisms of action.
